# Complete genome sequence of *Pseudomonas veronii* strain OST1911 isolated from oil sand tailing pond water in Alberta, Canada

**DOI:** 10.1128/MRA.00646-23

**Published:** 2023-11-15

**Authors:** Steven M. Shideler, Kira Goff, Jeff Gauthier, Roger C. Levesque, Shawn Lewenza

**Affiliations:** 1 Department of Immunology and Infectious Diseases, University of Calgary, Alberta, Canada; 2 Faculty of Science and Technology, Athabasca University, Athabasca, Alberta, Canada; 3 Institute of Integrative and Systems Biology, Laval University, Quebec, Canada; Loyola University Chicago, Chicago, Illinois, USA

**Keywords:** oil sands, bitumen, *Pseudomonas*

## Abstract

Here, we report the complete genome sequence of *Pseudomonas veronii* strain OST1911, recovered from oil sand process-affected water accumulated in tailing ponds. This water contains numerous organic and inorganic compounds of environmental significance. The genome size is 6,435,955 bp with a G+C content of 61.21%.

## ANNOUNCEMENT

Industrial extraction of heavy oil from bitumen results in the production of oil sand process-affected water (OSPW) that is contained within on-site ponds. A diverse community of bacteria survives within OSPW (1) despite the accumulation of polycyclic aromatic hydrocarbons, naphthenic acids, and BTEX (benzene, toluene, ethyl benzene, and xylenes) (2). Understanding microbial survival in OSPW is critical for developing bioremediation strategies. One such strain, *Pseudomonas veronii* OST1911, was isolated from OSPW samples obtained from the Athabasca oil sand region, plated on Pseudomonas isolation agar (BD Difco), and incubated at room temperature for 48 h. Purified single colonies were used for DNA isolation and long-term storage.

Genomic DNA was purified from an overnight pure culture in brain heart infusion (BHI) broth (BD Difco) using the DNeasy blood and tissue kit (Qiagen, Hilden, Germany) and then subjected to 40 s of mechanical fragmentation with a Covaris M220 ultrasonicator (Woburn, MA, USA). Library synthesis was performed with the KAPA HyperPrep kit (Kapa Biosystems, Wilmington, MA, USA), and barcoding was done with TruSeq HT adapters (Illumina, San Diego, CA, USA). An Illumina MiSeq 2  ×  300 bp paired-end run was used to obtain short reads as part of the International *Pseudomonas* Consortium Database (https://ipcd.ibis.ulaval.ca) (3).

Long reads were obtained via Oxford Nanopore MinION sequencing with an R9.4.1 flowcell. DNA from Luria Broth-grown pure cultures was obtained by phenol-chloroform-isoamyl extraction. MinION library preparation was done with the ligation sequencing kit (SQK-LSK109); sequencing was performed with MinKNOW v.19.12.2. Raw signal (fast5) was basecalled using Guppy v.3.4.1 and demultiplexed using qcat v.1.1.0 (4).

Illumina short reads (996,665 raw reads; average read length: R1 266.5 bp, R2 267.4 bp) were quality-controlled with FastQC v.0.11.9 (5), and Cutadapt v.2.8 (6) was used to remove adapter sequences and trim ends below a quality score of 20. This resulted in 967,638 paired-end reads with mean lengths of 226.9 bp (R1) and 197.2 bp (R2), providing 32× sequencing coverage. Quality control of MinION long-read sequences was done using Filtlong v.0.2.0 (https://github.com/rrwick/Filtlong) with the filtered Illumina short reads as a reference. Filtlong selected the best 85% reads above 1,000  bp matching the Illumina short reads. This resulted in 29,695 sequences with an average length of 7,607  bp, providing 35× sequencing coverage. The hybrid genome assembly of the short- and long-read sequences was performed with the Unicycler assembly pipeline v.0.4.8 (7). A complete, single-contig, circularized genome sequence was assembled with a size of 6,435,955  bp and a G + C content of 61.21% (as determined with QUAST v.5.0.2) (8). The publicly available genome sequence was annotated with PGAP v.4.13 (9).

Identification was done via whole-genome digital DNA-DNA hybridization (dDDH) using the Type Strain Genome Server pipeline (10). Based on whole-genome comparisons, the closest match was *Pseudomonas veronii* DSM 11331 (GenBank GCF_001439695.1). The dDDH value between *P. veronii* DSM 11331 and OST1911 was 85.3% (confidence interval 81.6% to 88.4%, species threshold 70%), indicating that OST1911 is a closely related strain of *P. veronii* DSM 11331.

## Data Availability

This hybrid assembly has been deposited in GenBank (CP129402) and raw reads in the NCBI sequence read archive (Illumina: SRR25130032 and Nanopore: SRR25130033), all under BioProject PRJNA325248.
